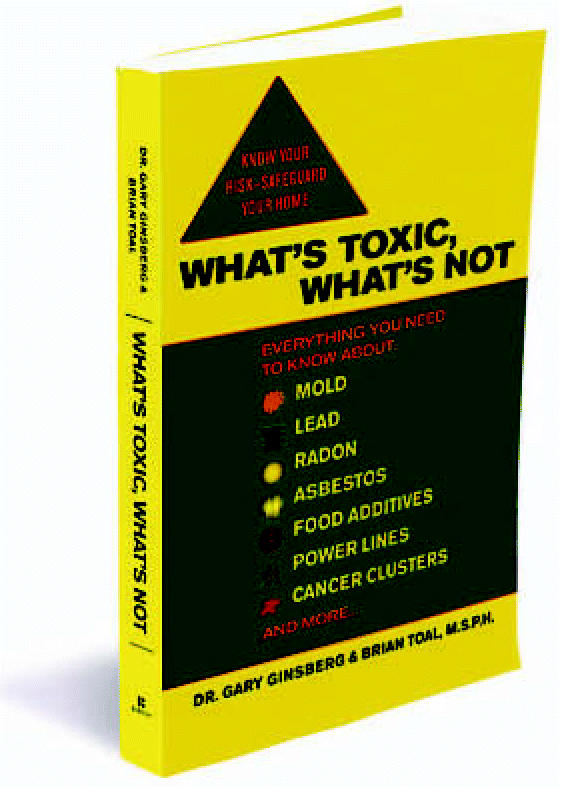# What’s Toxic, What’s Not

**Published:** 2007-01

**Authors:** David O. Carpenter

**Affiliations:** David O. Carpenter is the former dean of the School of Public Health, and is currently the director of the Institute for Health and the Environment at the University at Albany. His major area of research is human health effects of environmental contaminants

By Gary Ginsberg and Brian Toal

New York:Berkeley Publishing Group, 2006. 384 pp. ISBN: 0-425-21194-0, $15

*What’s Toxic, What’s Not* is a great book and one that fills an unmet need for accurate information presented in a nontechnical fashion. This book is written for the general public. The goal is to present information about environmental hazards in the home, the workplace, the school, and the community in a language that is easily understood. The book deals with hazards that are commonly recognized as well as some for which the public is, in general, not well informed.

The book is organized around toxicants at home and work (lead, radon, mold, asbestos), toxicants we eat, drink, or buy at the store (toxicants in food, water pollution, consumer products), toxicants in air (indoor and outdoor), and toxicants in the yard and neighborhood (garden, power lines, hazardous waste sites, volatile organics), and the authors also provide an informative section on cancer clusters.

Ginsberg and Toal use several tools very effectively in presenting their message. They rank each toxicant in terms of toxicity, exposure, and risk on a scale of 0 to 100, and this helps put the relative risk of this diverse group of agents in perspective. This ranking is particularly valuable for what they call “toxic uncertainties,” such as fluoride in drinking water, electromagnetic fields (EMFs), phthalates, polybrominated diphenyl ethers, and perfluorooctane sulfonate, where exposure is widespread but where the magnitude of toxicity is still a matter of debate. The authors list a series of questions and helpful answers, such as “This job is toxic: who can I call?” with the answer being to start with your employer, and if that doesn’t work to go to the Occupational Safety and Health Association (OSHA) website or state occupational health unit. They list the top 10 toxic myths (for example, the myth is that mold is a sign of poor housekeeping) and the realities (in this case, mold will grow wherever there is moisture) for specific toxics. The end of each chapter provides advice on how to determine whether you are exposed and, if so, steps that can be taken to reduce exposure. These are very effective ways to communicate important information.

As with any text that tries to make things easily understood and simple, in some areas important information is lost. The table stating that there are no adverse health effects of lead < 10 μg/dL is not accurate, despite the current position of the Centers for Disease Control and Prevention, and the statement that only lead levels of 40 μg/dL are dangerous is even less correct. There is no mention of the dangers of lead to adults, only warnings that adults should not bring lead home from their jobs and expose their children. The section on fish consumption references studies that I and my colleagues have conducted on contaminants in farmed salmon, and mistakenly states that dioxins are the major contaminant of concern, whereas it is polychlorinated biphenyls, dieldrin, and toxaphene, not dioxin, that drive the fish consumption advisories. In addition, the book recommends one meal of farm-raised salmon per week for women and children, whereas our results indicate elevated cancer risk from one meal or even less per month. The excellent chapter on EMF mentions only high-voltage power lines, even though the most common exposure comes from distribution lines. And there is no discussion of radiofrequency (cell phone) EMFs, a subject of considerable current interest.

These are minor issues, however, in what is an excellent and very readable book on the toxics in our environment. This book should find a place in every home. When it doesn’t answer all of the questions, it provides reference to sources that will. It is a wonderful resource that physicians and environmental health personnel can provide to patients and the public for easily understood information on exposures of concern to the community.

## Figures and Tables

**Figure:**